# Dural and Multiple Brain Metastases From Basaloid Nasopharyngeal Carcinoma: Case Report and Literature Review

**DOI:** 10.3389/fonc.2021.665652

**Published:** 2021-07-14

**Authors:** Xiaotao Geng, Furong Hao, Guiyan Han, Yaping Zhang, Peiyan Qin

**Affiliations:** ^1^ Department of Radiation Oncology, Weifang People’s Hospital, Weifang, China; ^2^ Department of Pathology, Weifang People’s Hospital, Weifang, China; ^3^ Department of Radiology, Weifang People’s Hospital, Weifang, China

**Keywords:** nasopharyngeal carcinoma, brain metastasis, dural metastasis, radiotherapy, chemotherapy, immunotherapy, basaloid squamous cell carcinoma, basaloid nasopharyngeal carcinoma

## Abstract

**Background:**

Nasopharyngeal carcinoma is an endemic head and neck cancer in Southern China. The common metastases organs involve bone, lung, and liver. Metastases in the dura and at multiple locations in the brain after a diagnosis of nasopharyngeal carcinoma are extremely rare.

**Case Presentation:**

We present a case of a 66-year-old man who initially complained of nasal congestion, epistaxis, and hearing impairment. The biopsy of the nasopharynx lesion showed basaloid squamous cell carcinoma. Eight months after conventional therapy, the patient was admitted to our hospital again with the complaint of a headache. A PET/CT scan was performed, revealing multiple metastases. A biopsy of subcutaneous soft tissue from the right upper arm was consistent with the previous biopsy. Palliative chemotherapy was administered. Thereafter, the patient had sudden dysfunction of the right side of the body. MRI demonstrated dural and multiple brain metastases. The therapeutic regimen then consisted of whole-brain radiotherapy, anti-angiogenesis therapy, and immunotherapy.

**Conclusions:**

This case highlights the diagnosis and treatment of uncommon metastases of nasopharyngeal carcinoma. Clinicians should remain vigilant for metastases during the treatment and follow-up periods.

## Introduction

Nasopharyngeal carcinoma (NPC) is an epithelial cancer characterized by its distinct ethnic and geographic distribution. It is rare in most parts of the world, but it is endemic in Southern China, Southeast Asia, and Northern Africa (1). World age-standardized incidence of NPC were 3.0/100,000 and 0.4/100,000, respectively, in China and Western countries in 2018 (1). Local relapse and distant metastases remain the main failure patterns of NPC after treatment (2). The application of intensity-modulated radiotherapy has provided excellent locoregional control; only 10–20% of patients will experience locoregional recurrence after primary treatment (3). Despite this progress, distant failure has remained the most critical NPC treatment challenge, occurring in 65.5% of all treatment failures in one cohort study (4). The common metastatic sites of NPC include bone, lung, and liver, but brain metastasis is rare. Here, we report one patient with dural and multiple intracranial metastases after initial treatment.

## Case Presentation

**Figure 1:**
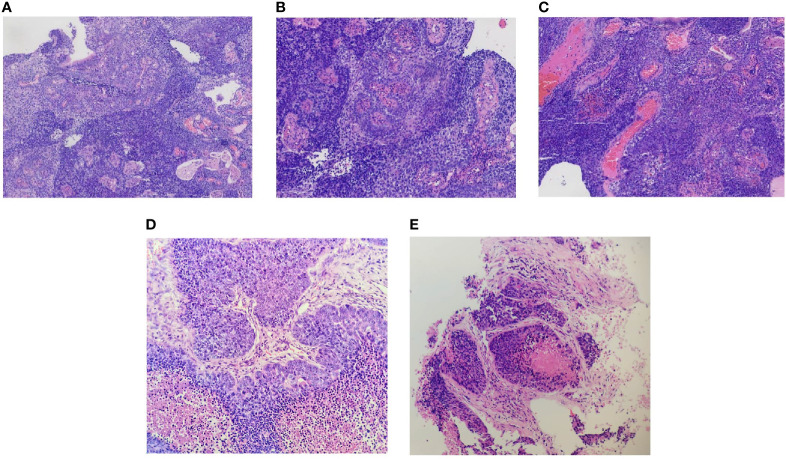
Biopsy of the primary nasopharyngeal neoplasm **(A–C)**, right upper arm lesion **(D)**, and lung metastases lesion **(E)**. **(A)** Epithelial papillary hyperplasia (hematoxylin and eosin, original magnification ×10). **(B)** Squamous differentiation (hematoxylin and eosin, original magnification ×20). **(C)** Base-like arrangement (hematoxylin and eosin, original magnification ×20). **(D)** Representative images of the metastatic carcinoma in the right upper arm (hematoxylin and eosin, original magnification ×20). **(E)** Representative images of the metastatic carcinoma in the lung (hematoxylin and eosin, original magnification ×20).

A 66-year-old man from Shandong Province, China, was admitted to our hospital on January 4, 2019, with the complaint of nasal congestion, epistaxis, and hearing impairment. He had a history of smoking for more than 50 years and drinking for more than 40 years. He had a left thoracic trauma and limb fracture in July 2018. MRI was performed and showed a large mass in the right posterior nasopharynx and right nasal cavity, invading the cavernous sinus. Retropharyngeal lymph nodes on the right side were also detected. A PET/CT scan was performed to exclude metastasis. Nasal endoscopy was performed, and a biopsy of the nasopharyngeal neoplasm confirmed basaloid squamous cell carcinoma (**Figures 1A–C**). Accordingly, the patient was diagnosed with basaloid squamous cell carcinoma of the nasopharynx (T4N1M0, stage IVA by the American Joint Committee on Cancer, 8th edition). He received two cycles of induction chemotherapy with docetaxel (75 mg/m^2^ on day 1) and cisplatin (75 mg/m^2^ on day 1). Thereafter, he went to another hospital and received radiotherapy combined with concurrent chemotherapy.

After experiencing migraines for one month, the patient was re-admitted to our hospital on September 11, 2019. A PET/CT scan revealed multiple fluorodeoxyglucose uptake and foci in the left parietal lobe, bilateral upper arm, left adrenal gland, mesentery, right ilium, and sacrum. We performed a biopsy of the right upper arm lesion (**Figure 1D**). The biopsy was positive for basaloid squamous cell carcinoma. Given his multiple metastases, the patient received palliative chemotherapy consisting of gemcitabine (1,000 mg/m^2^ on days 1 and 8).

**Figure 2:**
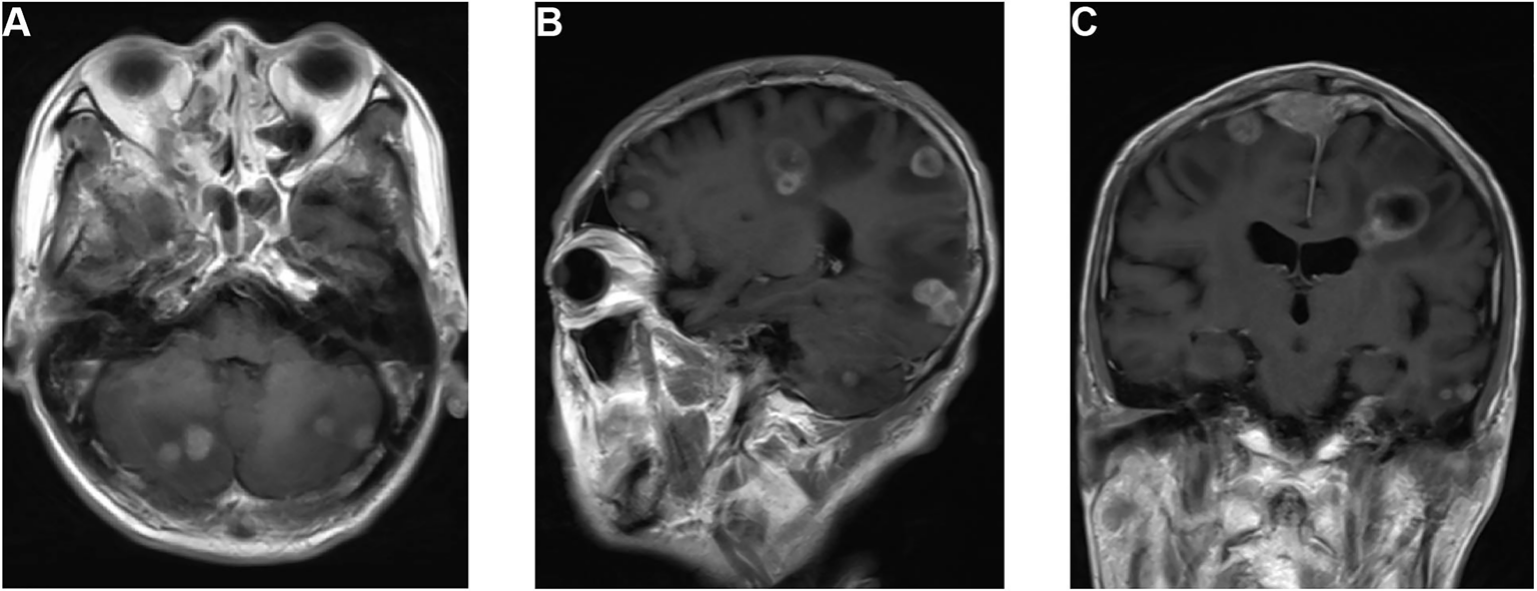
MRI of the dural and multiple brain metastases. **(A)** T1-weighted axial image shows cerebellum lesions. **(B)** T1-weighted sagittal image shows lesions in the cerebellum, occipital lobe, parietal lobe, and frontal lobe. **(C)** T1-weighted coronal image shows dural metastasis and lesions in the parietal and temporal lobes.

On September 29, 2019, the patient suddenly had dysfunction of his right body and fell down when going to the toilet. A CT scan was performed and showed multiple lesions in the brain. MRI was then performed and revealed dural metastasis and multiple intracranial metastatic lesions throughout both cerebral hemispheres and the cerebellum, including the frontal lobe, parietal lobe, and occipital lobe (**Figure 2**). Whole-brain intensity-modulated radiotherapy was initiated with a total dose of 40 Gy in 20 fractions. Meanwhile, to exclude primary lung malignancy, a biopsy of a lung lesion was performed, which showed that the pathology was consistent with results of the right upper arm and nasopharynx biopsy (**Figure 1E**); the metastatic lung lesions revealed progressive disease. Given the patient’s brain edema and rapid disease progression, he was treated with bevacizumab (5 mg/kg on day 1), anlotinib (10 mg on days 1–14), and toripalimab (240 mg on day 1) during radiotherapy. Later, the patient felt the pain near the sacrum and received palliative intensity-modulated radiotherapy to this site. Despite these comprehensive therapies, the disease continued to progress. On October 30, 2019, the patient died as a result of multiple organ failures.

## Discussion

The treatment failure pattern of NPC includes locoregional and distant metastases. Generally accepted treatment paradigms involve intensity-modulated radiotherapy, and only 10–20% of patients will experience locoregional recurrence after primary treatment. In contrast, distant metastasis has become the most common NPC failure pattern (4–6). Skeletal, pulmonary, and liver involvement are the top three locations of distant metastases of NPC after initial treatment (7). Although intracranial extension is common in locally advanced NPC, dissemination *via* blood vessels or cerebrospinal fluid to intracranial metastases is a conspicuous rarity. Lung cancer, breast cancer, and melanoma are the most frequent primary cancers to develop brain metastases; these three account for 67–80% of all metastatic brain tumors (8). Compared with these cancers, NPC’s progression to brain metastasis is rare, and it has been reported in case reports. To the best of our knowledge, there have been no reports of dural and multiple brain metastases of basaloid NPC combined with subcutaneous soft tissue involvement and multiple organ involvement.

A PubMed search of the English-language literature that reported brain metastases from NPC is summarized in **Table 1**. Most patients in these reports had intracranial metastases lesions located in the cerebral hemisphere, including the occipital lobe, temporal lobe and frontal lobe. Cerebellar metastasis was found in only one patient. What is the cause of this unbalanced distribution? The distribution of intracranial metastases follows roughly the relative weight and blood flow to each area (18). In addition, most patients had isolated lesions rather than multiple lesions. Only one patient had multiple lesions throughout both cerebral hemispheres, and one other patient had dural metastasis. To our knowledge, our report demonstrates the first case of dural and multiple brain metastases from NPC. A recent study reported a substantial accumulation of frontal lobe metastases from skin cancer and a cerebellar location of metastasis from breast and gastrointestinal cancer, which indicate the heterogeneous distribution of brain metastases (19). Ngan et al. (10) speculated that a microenvironment probably exists that helps circulating NPC cells settle into the occipital lobe. Because of the lack of case reports about NPC metastases, their distribution warrants additional exploration.

**Table 1:** Summary of previous studies reporting brain metastases from NPC.

First author (reference)	Year	Gender/age (years)	Nasopharynx histology	Location	Other organs involved	Treatment
Liaw et al. (9)	1994	Male/69	NKSSC	Bilateral occipital lobe	Bone	Chemotherapy + radiotherapy
Ngan et al. (10)	2002	Male/33	Poorly differentiated NPC	Left occipital lobe	Lung	Surgery + chemotherapy
Ozyar et al. (11)	2004	Male/41	Undifferentiated carcinoma	Right temporal lobe	No	Surgery + radiotherapy
Kaidar et al. (12)	2012	Male/54	Poorly differentiated NPC	Occipital lobe	Lung	Radiotherapy
Kuo et al. (13)	2014	Female/37	NKSSC	Left frontotemporal lobe	No	Surgery + chemotherapy + radiotherapy
Rafee et al. (14)	2014	Unknown/36	Unknown	Unknown	Spinal canal	Radiotherapy + chemotherapy + targeted therapy
Shen et al. (15)	2017	Female/47	NKSSC	Frontal lobe	Spinal cord	Chemotherapy + radiotherapy
		Male/47	NKSSC	Frontal lobe	Lung, bone, liver	Surgery + chemotherapy
Su et al. (16)	2019	Male/43	NKSSC	Unknown	Bone	Radiotherapy + chemotherapy
Park et al. (17)	2019	Female/49	Unknown	Right frontal lobe	Unknown	Radiotherapy
		Male/44	Unknown	Cerebellum	Unknown	Radiotherapy

NKSSC, non-keratinizing squamous cell carcinoma; NPC, nasopharyngeal carcinoma.

NPC is a rare malignancy in Western countries, with an incidence of 0.5–2 per 100,000 (12). However, the incidence of NPC is relatively high in southern China: 20 per 100,000 (20). There is no related reporting about the incidence of brain metastasis from NPC among Western countries and China. Our hospital is the largest in Weifang City, a third-tier city of Northern China. Since 2008, 316 patients with NPC have been admitted to our institution, and the incidence of brain metastasis from NPC in our institution is 0.3%. The epidemiology of brain metastasis from NPC must be explored through multiple institutions or in national cancer registries in the future.

According to the 4th Edition of the World Health Organization Classification of Head and Neck Tumours: Nasopharynx, there are three subtypes of NPC: keratinizing squamous cell carcinoma (KSCC), non-keratinizing squamous cell carcinoma (NKSCC), and basaloid squamous cell carcinoma (BSCC) (21). Brain metastases with NKSCC have been reported in five cases, and no brain metastases have beenreported with KSCC. We hypothesized that, according to published studies, NKSCC might be more predisposed than KSCC or BSCC to develop brain metastasis. The incidences of KSCC and BSCC are lower than that of NKSCC, so this hypothesis requires additional studies for confirmation. Our research is the first to report on brain metastases from BSCC. BSCC is considered an aggressive subtype, a variant of squamous cell carcinoma. This subtype most commonly occurs in the larynx, base of tongue, hypopharynx, and esophagus (22). The nasopharynx as a site of basaloid squamous cell carcinoma is extremely rare; few cases have been demonstrated in the literature (23–25). Its prognosis differs from that of other pathologic subtypes. A population-based study obtained from the surveillance, Epidemiology, and End Results (SEER) database has shown that this subtype has poorer 10-year survival compared with NKSCC (26).

Most studies report about brain metastases and other involved organs and often apply a treatment regimen of brain radiotherapy plus chemotherapy. Unlike patients in previous studies, the patient in our study had subcutaneous soft tissue and multiple organ involvement, and the disease progressed very quickly. Because of the numerous distant metastases, chemotherapy combined with immunotherapy and targeted therapy was included in the treatment. Immunotherapy, especially immune checkpoint inhibitors, has shown preliminary evidence of promising outcomes in patients with brain metastases from lung cancer and melanoma (27). We also tried immunotherapy; we administered toripalimab, a monoclonal antibody targeting programmed cell death protein 1. The targeted therapy we used was anlotinib (AL3818), a novel receptor tyrosine kinase inhibitor targeting vascular endothelial growth factor receptors 2 and 3, fibroblast growth factors 1–4, platelet-derived growth factor receptors α and β, c-Kit, and Ret; we also treated with bevacizumab. Though we used comprehensive regimens, the patient experienced disease progression. However, radiotherapy combined with immunotherapy or targeted therapy probably offers an alternative treatment for patients with brain metastases.

## Conclusions

Dural and multiple brain metastases from NPC are rare. This report highlights the experience of a patient with basaloid nasopharyngeal carcinoma that progressed to dural and multiple brain metastases. Clinicians must remain attentive to the risk of metastases after initial treatment of NPC and stay vigilant for rare manifestations. In this case, because the efficacy of the comprehensive treatment plan was not satisfactory, we offered an alternative regimen that included radiotherapy, immunotherapy, and targeted therapy to treat the brain metastases. Additional study to investigate the epidemiology, distribution, differences among three histological subtypes of brain metastases from NPC and determine the appropriate treatment is warranted.

## Data Availability Statement

The original contributions presented in the study are included in the article/supplementary material. Further inquiries can be directed to the corresponding author.

## Ethics Statement

The studies involving human participants were reviewed and approved by the Ethics Committee of Weifang People’s Hospital. Written informed consent for participation was not required for this study in accordance with the national legislation and the institutional requirements.

## Author Contributions

XG retrieved clinical data and wrote and edited the manuscript. FH supervised the writing. GH captured biopsy images and assisted with figure development. YZ captured MRI images and assisted with figure development. PQ conceived this article, retrieved clinical data, and assisted with editing the manuscript. All authors contributed to the article and approved the submitted version.

## Funding

This work was supported by the Science and Technology Development Project of Weifang City (Grant No. 2019YX003).

## Conflict of Interest

The authors declare that the research was conducted in the absence of any commercial or financial relationships that could be construed as a potential conflict of interest.
